# Evaluation of the Nrf2–Keap1 and Sestrin-2 Pathways in the Serum of Patients with Hidradenitis Suppurativa †

**DOI:** 10.3390/medicina62081611

**Published:** 2026-08-21

**Authors:** Ismail Hakki Gurbuz, Idris Demir, Serhat Inaloz, Muhammed Enes Taysi, Seyithan Taysi

**Affiliations:** 1 Department of Medical Biochemistry, Faculty of Medicine, Gaziantep University, 27410 Gaziantep, Turkey; ishakbayram1971@gmail.com; 2 Department of Dermatology, Faculty of Medicine, Gaziantep University, 27410 Gaziantep, Turkey; dridrisdemir@gmail.com (I.D.); serhatinaloz@hotmail.com (S.I.); 3 Division of Emergency Medicine, Cankiri State Hospital, 18100 Cankiri, Turkey; enestaysi65@gmail.com; 4 Phytotherapy and Medicinal-Aromatic Plants Application and Research Center, Gaziantep University, 27410 Gaziantep, Turkey

**Keywords:** hidradenitis suppurativa, Nrf2, Keap1, Sestrin-2, oxidative stress, biomarkers, matrix metalloproteinases

## Abstract

*Background and Objectives*: Hidradenitis suppurativa (HS) is a chronic inflammatory skin disease characterised by recurrent nodules, abscesses, sinus tract formation, and tissue remodelling. Increasing evidence suggests that oxidative stress contributes to HS pathogenesis; however, the role of the Nrf2–Keap1–Sestrin-2 axis and its interaction with matrix remodelling pathways remains poorly understood. This study aimed to evaluate serum levels of Nrf2, Keap1, Sestrin-2, asprosin, MMP-1, and TIMP-1 in patients with HS and to investigate their potential roles in disease pathophysiology. *Materials and Methods*: A total of 26 patients with hidradenitis suppurativa and 20 healthy volunteers were enrolled in the study. Serum biomarkers were measured in patients with HS and healthy controls. In addition to conventional statistical analyses, multivariate analyses, including PCA, PLS-DA, VIP scoring, correlation mapping, ROC analysis, heatmap visualisation, and biplot assessment, were performed to characterise biomarker interactions and discriminatory performance. *Results*: HS patients exhibited significantly increased serum asprosin, Keap1, and MMP-1 levels, whereas Nrf2, Sestrin-2, and TIMP-1 levels were significantly reduced compared with controls. Multivariate analyses demonstrated clear separation between patient and control groups within the cohort, indicating a distinct biochemical signature associated with HS. VIP analysis identified Nrf2, Sestrin-2, and TIMP-1 as the most influential variables contributing to group discrimination. Within the HS group, none of the pairwise biomarker correlations remained statistically significant after Benjamini–Hochberg false discovery rate (FDR) correction. ROC analysis showed diagnostic performance for Nrf2 and Sestrin-2. *Conclusions*: These findings suggest that alterations in serum markers related to the Nrf2–Keap1–Sestrin-2 axis and the MMP-1/TIMP-1 balance are associated with oxidative stress, inflammation, and tissue remodelling in HS. Nrf2 and Sestrin-2 may represent candidate biomarkers; however, given the modest sample size and single-centre design, their discriminatory performance should be considered exploratory and requires confirmation in larger independent cohorts.

## 1. Introduction

Hidradenitis suppurativa (HS), a chronic inflammatory disease characterized by painful, deep-seated nodules, abscesses, and drainage tunnels in the skin of the armpits, groin, genital and anal areas, or under the breasts, has seen significant advances in understanding in recent years. This condition typically begins in the second or third decade of life. HS prevalence is estimated to range globally from 0.1% to 4%; there is a marked female predominance, and onset usually occurs after puberty [1,2]. Despite its recognition as a distinct dermatological entity, the pathogenesis of HS remains incompletely understood, and the role of genetic factors is unclear.

Traditionally, HS was considered a follicular occlusion disorder, initiated by infundibular hyperkeratosis and rupture of the hair follicle wall. However, current evidence supports its classification as a systemic autoinflammatory disease, characterized by dysregulated innate and adaptive immune responses [3]. Dysregulated expression of pro-inflammatory cytokines, including tumor necrosis factor-α (TNF-α), interleukin (IL)-1β, IL-17, and IL-23, has been reported in HS, particularly in lesional tissue, with systemic alterations also described [4]. These mediators promote neutrophil recruitment, macrophage activation, and keratinocyte proliferation, perpetuating chronic inflammation and tissue destruction. Beyond immunological dysregulation, increasing attention has focused on the pivotal role of oxidative stress in HS pathophysiology [2,5].

Oxidative stress, defined as an imbalance between reactive oxygen species (ROS) production and antioxidant defences [6,7], contributes to chronic inflammation, lipid peroxidation, and cellular dysfunction across various dermatological diseases. In HS, persistent inflammation drives excessive ROS generation by neutrophils, macrophages, and keratinocytes, leading to oxidative injury to proteins, lipids, and nucleic acids. This redox imbalance exacerbates immune activation, promotes extracellular matrix degradation, and impairs wound healing. Indeed, several studies have reported elevated oxidative stress biomarkers, including malondialdehyde (MDA) and 8-isoprostanes, and reduced levels of antioxidant enzymes, such as superoxide dismutase (SOD) and glutathione peroxidase (GSH-Px), in patients with HS [8,9,10].

The nuclear factor erythroid 2-related factor 2 (Nrf2) pathway is a critical endogenous defence against oxidative stress. Nrf2 is a transcription factor that regulates the expression of more than 200 cytoprotective genes involved in antioxidant defence, detoxification, and metabolic balance [11]. Under basal conditions, Nrf2 is sequestered in the cytoplasm by its inhibitor, Kelch-like ECH-associated protein 1 (Keap1), which promotes its ubiquitination and degradation via the ubiquitin–proteasome system. Upon exposure to oxidative or electrophilic stress, Nrf2 dissociates from Keap1, translocates to the nucleus, and binds to antioxidant response elements (AREs), thereby inducing transcription of genes such as heme oxygenase-1 (HO-1), NAD(P)H quinone dehydrogenase 1 (NQO1), and glutathione S-transferases (GSTs) [12,13]. Impaired activation of the Nrf2 pathway has been implicated in chronic inflammatory disorders, including psoriasis, atopic dermatitis, and rheumatoid arthritis [13,14]. Nevertheless, its role in HS remains poorly characterised.

Parallel to Nrf2 signalling, the Sestrin-2 protein—a highly conserved, stress-inducible antioxidant—plays a crucial role in cellular adaptation to oxidative and metabolic stress. Sestrin-2 modulates redox balance by suppressing ROS accumulation and regulates autophagy by inhibiting the mammalian target of rapamycin complex 1 (mTORC1) pathway. Moreover, Sestrin-2 expression is transcriptionally regulated by both p53 and Nrf2, forming an integrated network that coordinates the cellular stress response [11,12]. Reduced Sestrin-2 expression has been linked to heightened oxidative stress, mitochondrial dysfunction, and inflammatory signalling in diseases such as diabetes, obesity, and cardiovascular disorders [13,14]. Given the systemic inflammatory and metabolic alterations observed in HS, dysregulation of the Nrf2–Keap1–Sestrin-2 axis may be a key contributor to disease progression.

Another relevant molecular component in HS pathogenesis is the imbalance between MMP-1 and its endogenous inhibitor, TIMP-1. MMP-1 is a zinc-dependent proteolytic enzyme responsible for extracellular matrix (ECM) turnover, wound remodelling, and tissue repair [15]. Overactivation of MMP-1, coupled with reduced TIMP activity, leads to excessive ECM degradation, tissue destruction, and sinus tract formation—core features of HS pathology. Notably, oxidative stress has been shown to upregulate MMP-1 and suppress TIMP expression via redox-sensitive transcription factors such as NF-κB and activator protein-1 (AP-1) [16]. Therefore, the interplay among oxidative stress, antioxidant signaling (Nrf2/Sestrin-2), and protease activity (MMP/TIMP) may represent an integrated pathogenic network underlying HS lesions.

In addition to oxidative and proteolytic dysregulation, recent studies have highlighted the potential role of metabolic hormones in HS. Asprosin, a fasting-induced glucogenic hormone secreted by white adipose tissue, has emerged as a modulator of glucose homeostasis and systemic inflammation [17]. Elevated asprosin levels have been linked to insulin resistance, obesity, and metabolic syndrome—conditions that are frequently comorbid with HS. Accumulating evidence suggests that elevated asprosin levels are associated with chronic inflammation, insulin resistance, and increased oxidative stress. Experimental studies indicate that asprosin may stimulate ROS generation through NOX-dependent pathways and pro-inflammatory cytokine signaling, thereby amplifying redox imbalance and tissue inflammation [18,19,20]. However, its relationship with Nrf2–Keap1 signaling and Sestrin-2 expression has not yet been explored.

Collectively, the growing evidence indicates that HS involves a complex interplay among oxidative stress, metabolic dysregulation, immune activation, and extracellular matrix remodeling. Despite advances in understanding its immunopathology, a critical gap remains in identifying molecular pathways that link these processes and could serve as both diagnostic biomarkers and therapeutic targets. Given the pivotal role of the Nrf2–Keap1–Sestrin-2 axis in regulating oxidative and inflammatory balance, dysregulation of this signaling network may contribute to the persistent oxidative stress and tissue destruction characteristic of HS. Therefore, this study aimed to evaluate serum levels of Nrf2, Keap1, and Sestrin-2, along with oxidative stress-related and matrix-remodeling markers (asprosin, MMP-1, and TIMP-1) in patients with hidradenitis suppurativa compared with healthy controls. By integrating these molecular parameters, this research sought to elucidate the potential pathophysiological role of the Nrf2–Keap1–Sestrin-2 signaling axis in HS and to explore its association with metabolic and structural pathways implicated in disease development. Understanding these interactions may not only shed light on HS pathogenesis but also identify novel biomarkers for disease monitoring and potential targets for antioxidant or metabolism-based therapeutic interventions.

## 2. Materials and Methods

### 2.1. Ethical Approval

This study was approved by the Clinical Research Ethics Committee of Gaziantep University under decision number 2023/160.

### 2.2. Participants and Eligibility Criteria

This cross-sectional study included 26 adults aged 18–65 years with a clinical diagnosis of hidradenitis suppurativa established by a dermatologist at the Dermatology Outpatient Clinic of Gaziantep University Faculty of Medicine. Recruitment was based on eligible patients evaluated during the study period and was not formally documented as consecutive sampling. The inclusion criteria were age 18–65 years, a clinical diagnosis of HS, and written informed consent. Exclusion criteria included pregnancy, breastfeeding, and prior biologic treatment. The control group consisted of 20 healthy volunteers aged 18–65 years with no known chronic diseases who were neither pregnant nor breastfeeding. No formal individual or frequency-matching procedure was applied. A separate publicly registered or preregistered statistical analysis plan was not available. Therefore, multivariate, correlation, and ROC analyses were considered exploratory.

### 2.3. Collection of Materials

Blood samples from patients diagnosed with hidradenitis suppurativa and from the control group were collected in anticoagulant-free tubes and centrifuged at 4000× *g* for 10 min to obtain serum. The serum samples were then placed in 4 Eppendorf tubes and stored at −80 °C until biochemical parameters were analyzed.

### 2.4. Measurement of Biochemical Parameters

Serum levels of Nrf2, Keap1, Sestrin2, Asprosin, MMP-1, and TIMP-1 were measured using an enzyme-linked immunosorbent assay (ELISA) (BT LAB, Jiaxing Korain Biotech Co., Ltd., Jiaxing, Zhejiang, China). Color intensity generated by the ELISA was measured with an ELISA reader (Biotek ELx800, BioTek Instruments. Inc, Winooski, VT, USA). Keap1 was expressed in ng/L and the other parameters in ng/mL. All procedures were performed at the Department of Medical Biochemistry, Gaziantep University Faculty of Medicine.

Serum concentrations of Nrf2, Keap1, Sestrin-2, asprosin, MMP-1, and TIMP-1 were measured using commercially available human sandwich ELISA kits from BT LAB, Jiaxing Korain Biotech Co., Ltd. (Jiaxing, China). The kits’ catalog numbers and analytical characteristics were as follows: Nrf2, E3244Hu, analytical range 0.2–60 ng/mL and sensitivity 0.11 ng/mL; Keap1, E5534Hu, analytical range 15–3000 ng/L and sensitivity 7.87 ng/L; Sestrin-2, E3437Hu, analytical range 0.05–15 ng/mL and sensitivity 0.01 ng/mL; asprosin, E4095hu, analytical range 0.5–100 ng/mL and sensitivity 0.23 ng/mL; MMP-1, E0916Hu, analytical range 0.05–70 ng/mL and sensitivity 0.01 ng/mL; and TIMP-1, E1236Hu, analytical range 5–1800 pg/mL and sensitivity 2.48 pg/mL. The manufacturer-reported intra-assay and inter-assay coefficients of variation were <8% and <10%, respectively, for all assays.

### 2.5. Statistical Analyses

Statistical analyses were conducted using SPSS Statistics version 27.0 (IBM Corp., Armonk, NY, USA). The normality of continuous variables was assessed using the Kolmogorov–Smirnov test. Normally distributed continuous variables were expressed as mean ± standard deviation (SD) and compared between groups using the independent-samples Student’s *t*-test. Non-normally distributed continuous variables were presented as median and interquartile range (IQR) and compared using the Mann–Whitney U test. Categorical variables were expressed as number and percentage [*n* (%)] and compared using Pearson’s chi-square test. Fisher’s exact test was used when the assumptions of the chi-square test were not met because of small expected cell counts. Associations between numerical variables were evaluated using Pearson’s correlation analysis for normally distributed variables and Spearman’s rank correlation analysis for non-normally distributed variables, as appropriate. To account for multiple testing, the Benjamini–Hochberg false discovery rate (FDR) procedure was applied separately to *p*-values obtained from between-group biomarker comparisons and correlation analyses. FDR-adjusted *p*-values were reported. For correlation analyses, associations with an unadjusted *p* < 0.05 were regarded as nominally significant, whereas an FDR-adjusted *p* < 0.05 was required for statistical significance after correction for multiple comparisons.

Receiver operating characteristic (ROC) curve analysis was performed to evaluate the ability of the investigated biomarkers to discriminate patients with hidradenitis suppurativa (HS) from healthy controls. The area under the ROC curve (AUC) was calculated for each biomarker. All statistical tests were two-tailed, and *p* < 0.05 was considered statistically significant unless otherwise specified by the FDR correction. Exploratory multivariate analyses were performed using MetaboAnalyst 6.0. Variable Importance in Projection (VIP) scores, correlation mapping, heatmap analysis, fold-change analysis, and biplot analysis were used to investigate the relative contributions, clustering patterns, and interrelationships of the evaluated biomarkers. Given the modest sample size, the multivariate and ROC analyses were considered exploratory and were interpreted accordingly.

## 3. Results

The available demographic and clinical characteristics are summarized in Table 1.

Data are presented as median (interquartile range [IQR]) or number (percentage), as appropriate. Age was compared between the patient and control groups using the Mann–Whitney U test. Categorical variables were compared using Pearson’s chi-square test or Fisher’s exact test when the assumptions of the chi-square test were not met. No between-group statistical comparison was performed for family history of HS or Hurley stage because these variables were applicable only to the patient group. *p* < 0.05 was considered statistically significant. * Indicates statistical significance.

Compared to the control group, serum asprosin, Keap1, and MMP-1 levels were statistically significantly higher in the HS patient group, while Nrf2, TIMP-1, and Sestrin2 levels were lower (Table 2).

**Figure 1:**
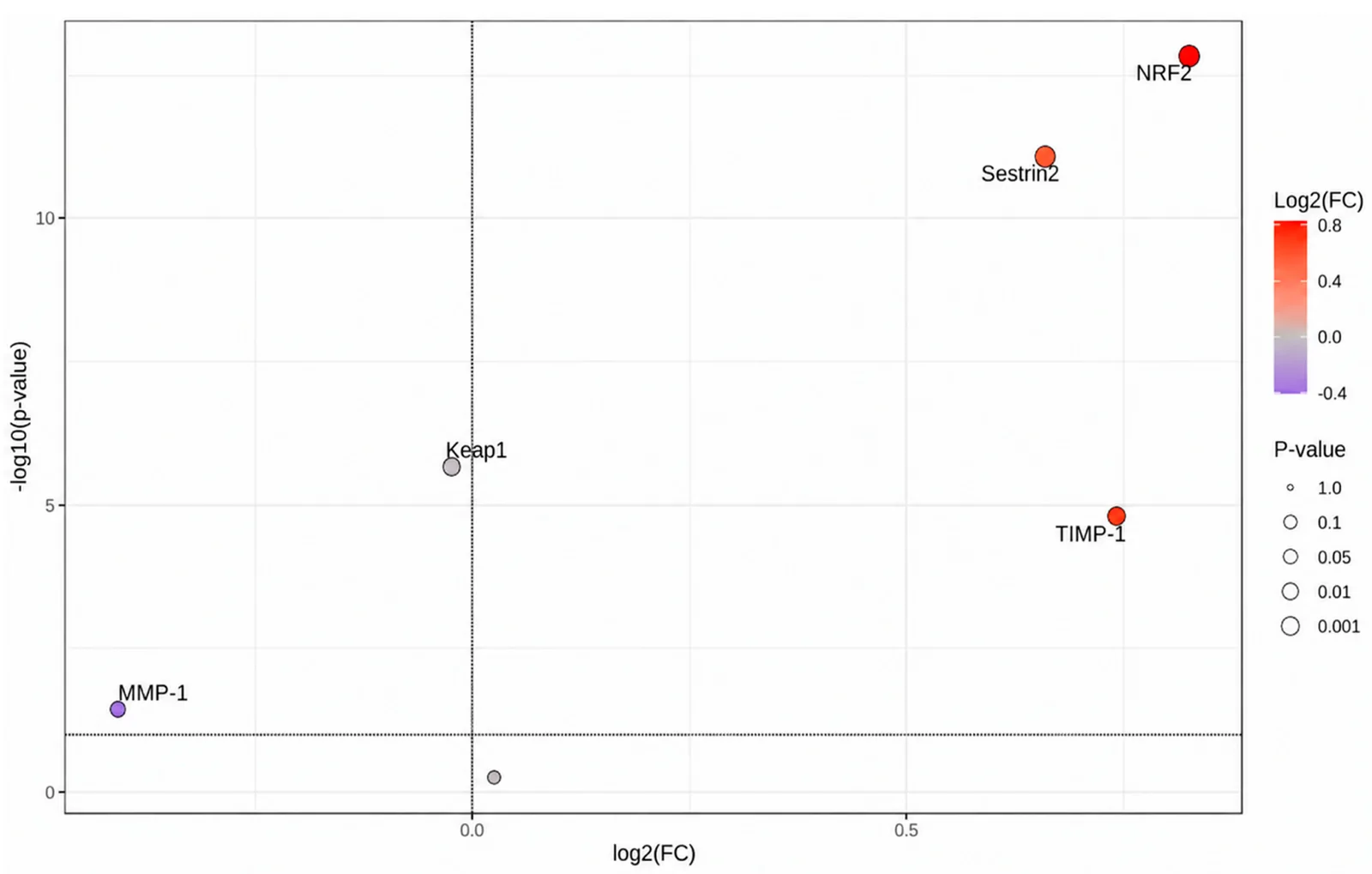
Fold-Change Analysis of Serum Biomarkers.

Fold-change plot of serum biomarkers in patients with hidradenitis suppurativa compared with healthy controls. Serum Keap1, asprosin, and MMP-1 levels were increased, whereas Nrf2, Sestrin-2, and TIMP-1 levels were decreased in the HS group (Figure 1).

**Figure 2:**
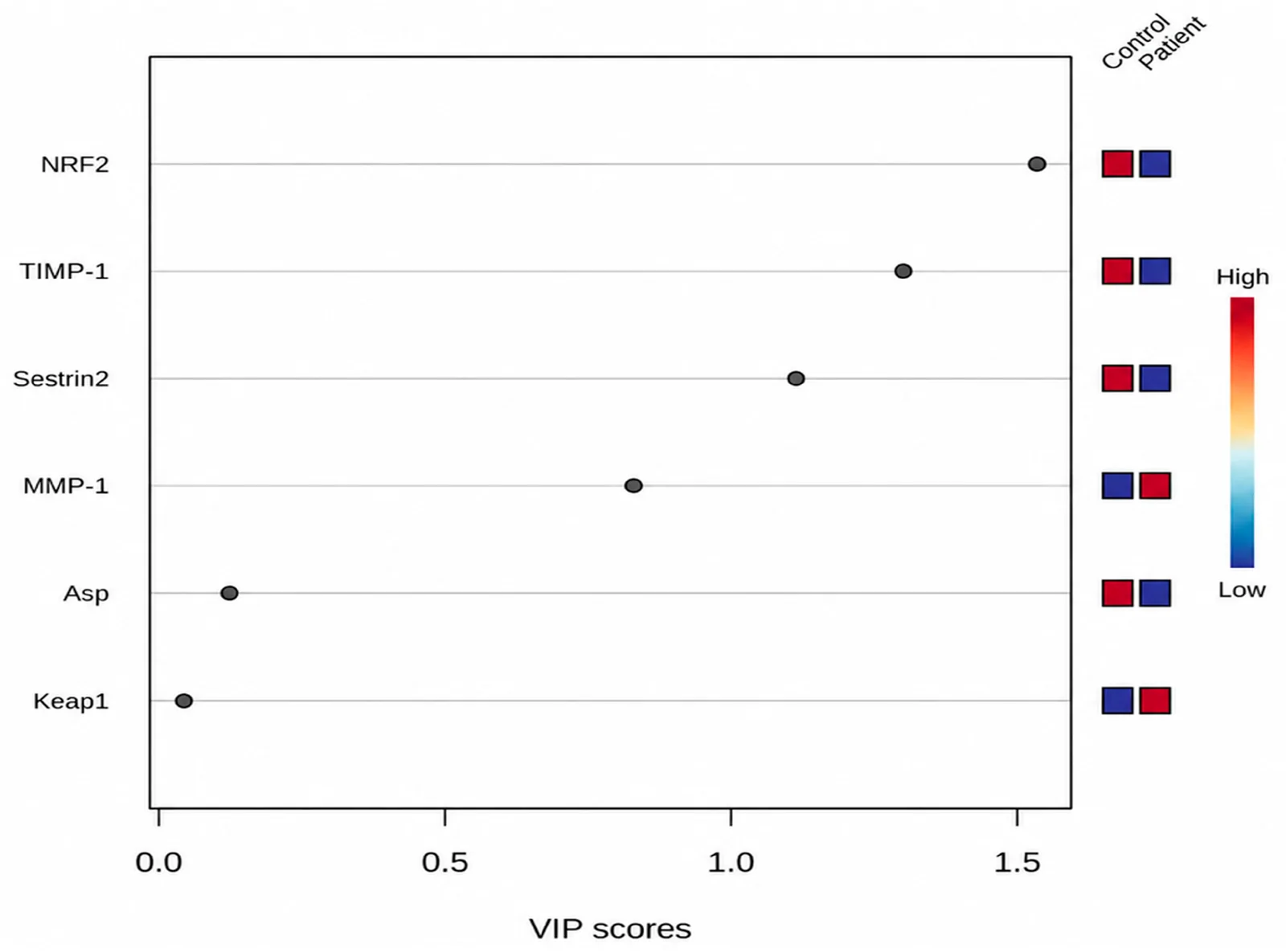
VIP Score. Asp: Asprosin.

In the present PLS-DA model, Nrf2, Sestrin-2, and TIMP-1 had the highest VIP scores and contributed most strongly to the observed separation between patients with HS and healthy controls. Keap1, asprosin, and MMP-1 had comparatively lower contributions to the model (Figure 2).

**Figure 3:**
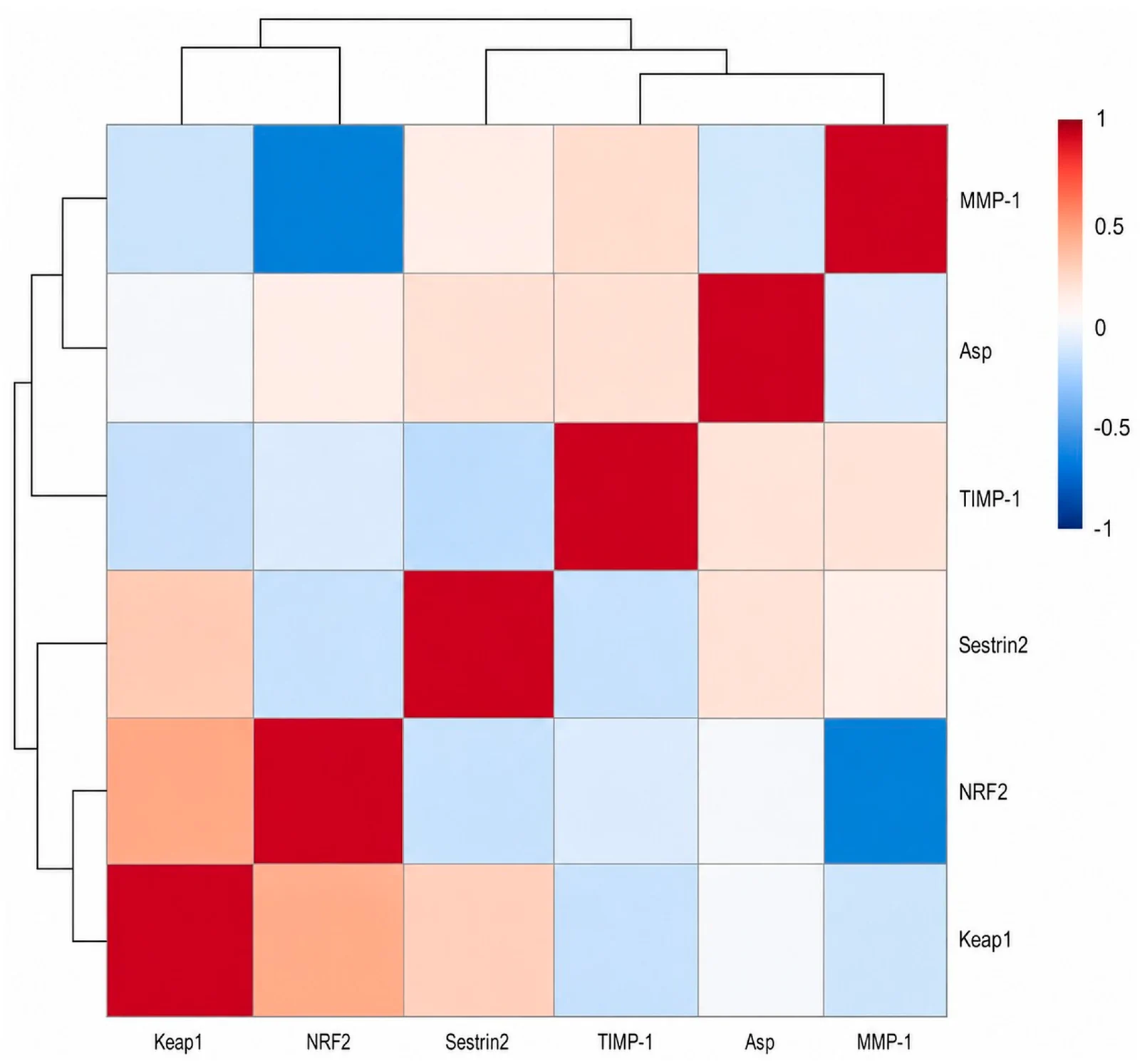
Correlation Map. Asp: Asprosin.

The correlation map illustrates the direction and magnitude of pairwise associations among the evaluated biomarkers within the HS group. Although Nrf2 showed nominal associations with Keap1 and MMP-1, none of the correlations remained statistically significant after FDR correction. Therefore, the map should be interpreted as an exploratory visualization of correlation patterns rather than evidence of statistically robust or mechanistic relationships (Figure 3).

Within the HS group, Nrf2 showed a nominal positive correlation with Keap1 (ρ = 0.465, raw *p* = 0.0168) and a nominal negative correlation with MMP-1 (ρ = −0.506, raw *p* = 0.0083). However, neither association remained statistically significant after Benjamini–Hochberg FDR correction (adjusted *p* = 0.1258 and 0.1246, respectively). No other pairwise correlations were statistically significant, and none of the evaluated correlations remained significant after FDR correction (Table 3).

**Figure 4:**
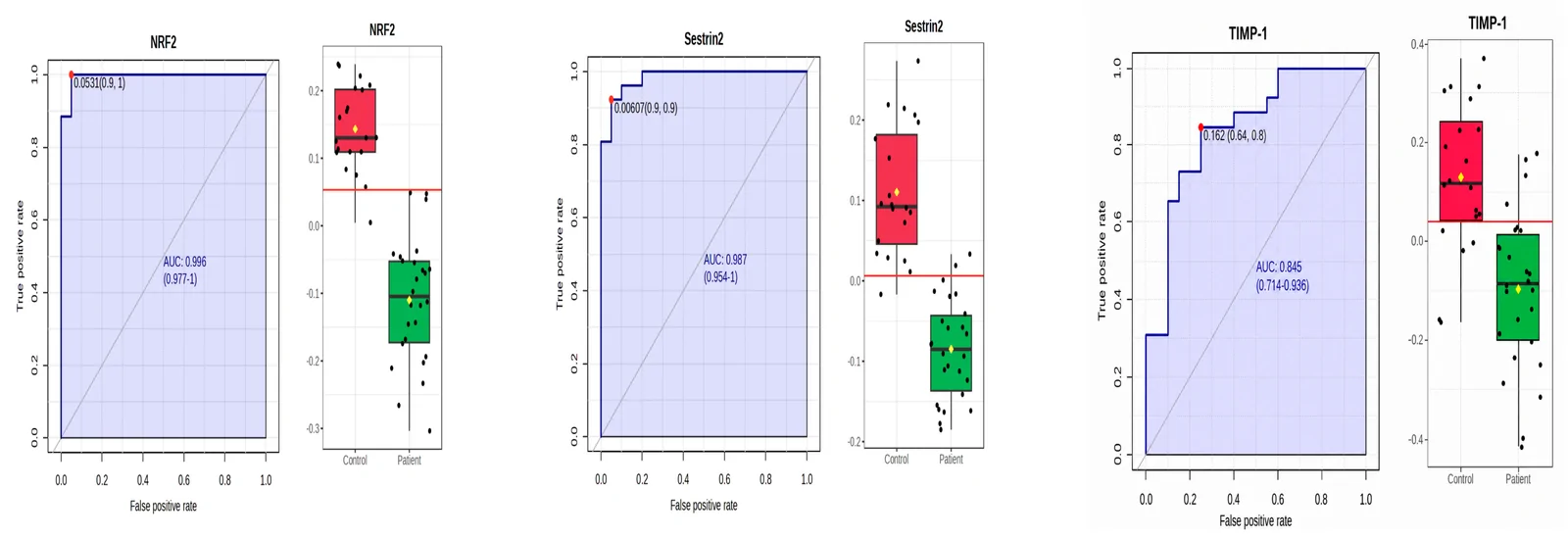
Evaluation of the Discriminatory Performance of Nrf2, Sestrin-2, and TIMP-1 in HS Diagnosis by ROC Analysis.

ROC analyses showed high apparent discriminatory ability for Nrf2 (AUC = 0.996) and Sestrin-2 (AUC = 0.987), whereas TIMP-1 showed comparatively lower performance (AUC = 0.846) in distinguishing patients with HS from healthy controls in the present cohort. However, because these estimates were derived from a small, single-center cohort without independent validation, they should be regarded as exploratory measures of group discrimination rather than evidence of disease specificity or established clinical diagnostic utility (Figure 4).

**Figure 5:**
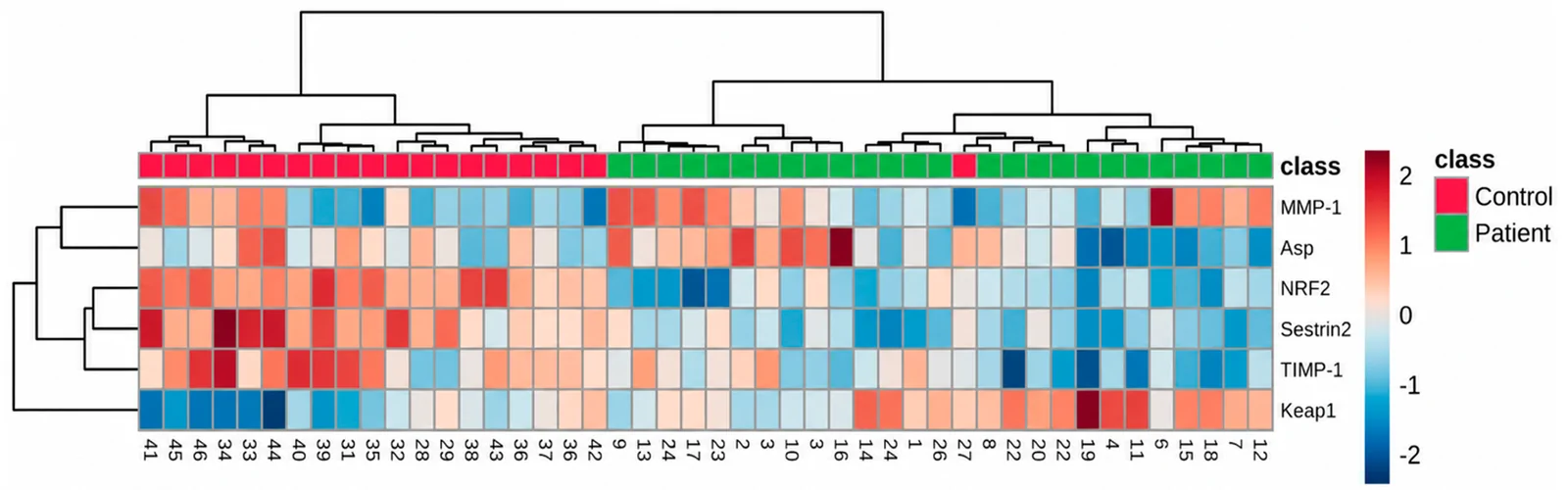
Heat Map Chart.

The heatmap showed relative differences in biomarker profiles between patients with HS and healthy controls. Nrf2, Sestrin-2, and TIMP-1 generally had lower standardized values in the HS group, whereas Keap1, asprosin, and MMP-1 generally had higher standardized values. The observed clustering pattern was consistent with the univariate group comparisons and should be regarded as an exploratory visualization of biomarker variation (Figure 5).

**Figure 6:**
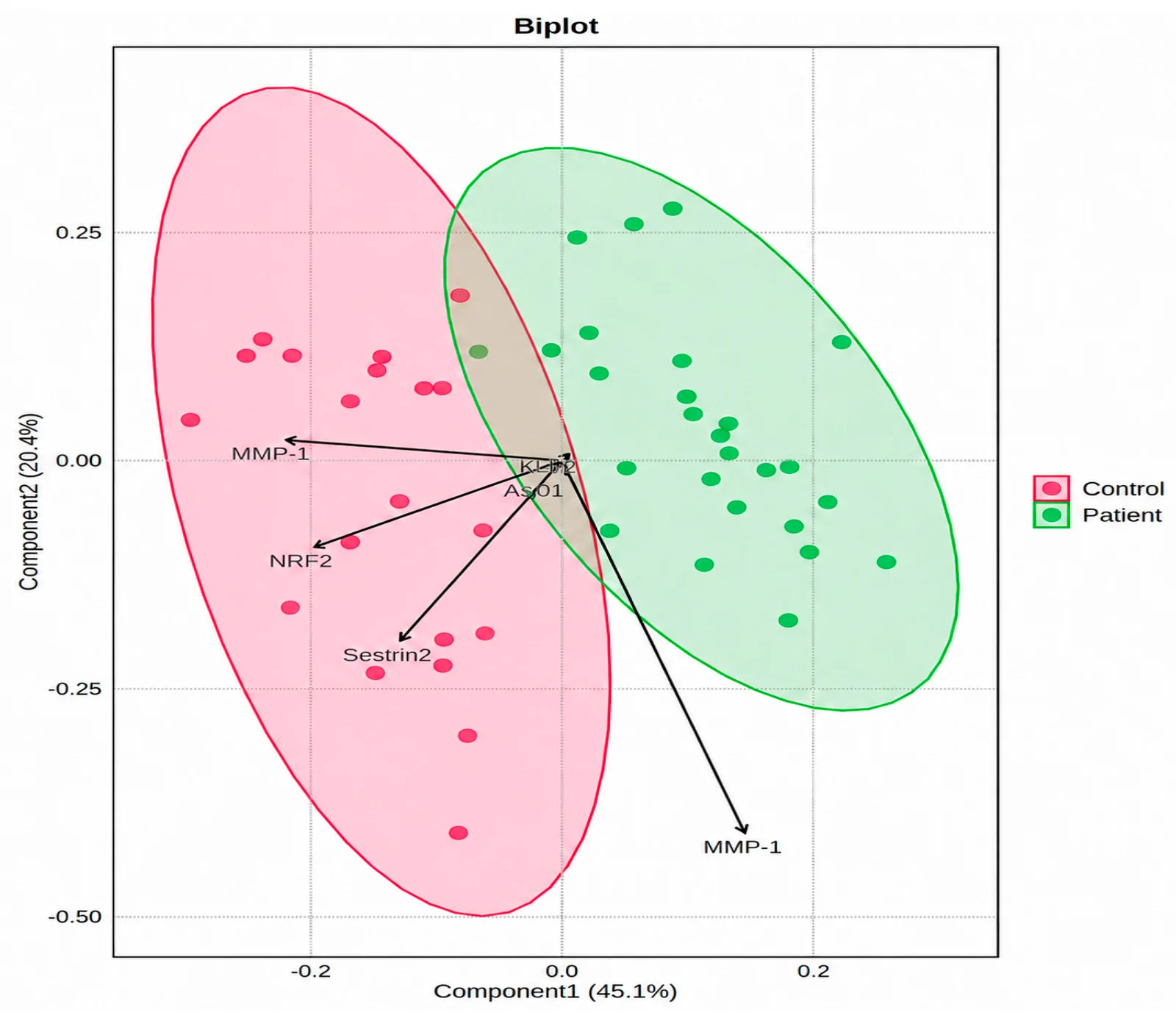
Biplot Chart. Asp: Asprosin.

The biplot illustrated the distribution of patients with HS and healthy controls, along with the relative orientations of the evaluated biomarkers. Nrf2, Sestrin-2, and TIMP-1 were oriented toward the control group, whereas Keap1, asprosin, and MMP-1 were oriented toward the HS group. The vector directions were consistent with the group differences observed in the univariate analyses; however, they represent associations with the principal components rather than causal or regulatory relationships (Figure 6).

## 4. Discussion

Hidradenitis suppurativa is a chronic, recurrent, and debilitating inflammatory skin disease characterized by painful nodules, abscesses, and sinus tract formation primarily in intertriginous areas. Although its exact etiology remains elusive, accumulating evidence suggests that HS is not merely an infectious condition but a systemic autoinflammatory disorder involving aberrant immune responses, oxidative stress, and dysregulated metabolic signaling [1,9,10]. This clinical study measured serum levels of asprosin, Keap1, Nrf2, sestrin-2, MMP-1, and TIMP-1 in patients with HS and compared them with those of healthy controls. The findings indicate that oxidative stress-related signaling pathways, particularly the Nrf2–Keap1 and Sestrin-2 systems, are significantly altered in HS and may play a central role in its pathophysiology and clinical manifestations.

Although Nrf2 and Keap1 were measurable in serum using commercially available immunoassays, both proteins predominantly function within intracellular signaling networks. Therefore, their circulating concentrations should not be considered direct measures of intracellular Nrf2–Keap1 pathway activity, Nrf2 nuclear translocation, or antioxidant response element-dependent transcription. Circulating immunoreactive Nrf2 and Keap1 may originate from cellular turnover or injury or be associated with extracellular vesicles or other protein complexes; however, their precise biological source and the possibility of active secretion remain uncertain. Because extracellular-vesicle fractions, paired lesional tissue, peripheral blood mononuclear cells, subcellular localization, and downstream Nrf2 targets were not evaluated, the present findings should be interpreted as altered circulating protein profiles associated with HS rather than direct evidence of intracellular pathway disruption [21,22].

In the study, serum asprosin, Keap1, and MMP-1 levels were significantly elevated in HS patients, whereas sestrin-2, TIMP-1, and Nrf2 levels were significantly reduced. These findings collectively support the hypothesis that an imbalance between oxidative stress and antioxidant defense contributes to the chronic inflammatory milieu observed in HS. The Keap1–Nrf2 axis is a pivotal cellular defense mechanism against oxidative injury. Under physiological conditions, Keap1 negatively regulates Nrf2 by promoting its ubiquitination and degradation. In response to oxidative stimuli, Nrf2 dissociates from Keap1, translocates to the nucleus, and induces the transcription of antioxidant and cytoprotective genes, including heme oxygenase-1 (HO-1), NAD(P)H quinone dehydrogenase 1 (NQO1), and glutathione peroxidases [8,22,23]. The significant reduction in Nrf2 levels observed in HS patients in this study suggests a compromised antioxidant response, which could perpetuate oxidative damage and inflammatory signaling.

The concomitant increase in Keap1 levels may be consistent with altered systemic redox regulation in HS. However, because Nrf2 and Keap1 were measured in serum, these findings should not be interpreted as direct evidence of altered intracellular Nrf2–Keap1 signaling or Nrf2 nuclear activity. Within the HS group, Nrf2 showed a nominal negative correlation with MMP-1 (ρ = −0.506, *p* = 0.0083); however, this association did not remain statistically significant after FDR correction (adjusted *p* = 0.1246). Therefore, although the direction of this association may warrant further investigation, the present data do not support a statistically robust or mechanistic relationship between circulating Nrf2 and MMP-1.

Sestrin-2 is a stress-inducible antioxidant protein regulated by p53 and Nrf2 that modulates oxidative stress and autophagy by inhibiting the mTORC1 pathway [24,25]. Serum Sestrin-2 levels were lower in patients with HS than in healthy controls, which may reflect an altered response to cellular stress. Sestrin-2 is known to limit ROS accumulation and contribute to redox homeostasis [11,26]. However, within the HS group, Sestrin-2 showed no statistically significant correlations with Nrf2, Keap1, asprosin, MMP-1, or TIMP-1, and none of these associations were significant after FDR correction. Therefore, the reduced circulating Sestrin-2 concentration observed in patients with HS should be interpreted primarily as a group-level biomarker difference rather than evidence of coordinated regulation or mechanistic interaction with the other circulating biomarkers evaluated in this study. Further studies incorporating tissue- or cell-based measurements are required to clarify the biological significance of altered circulating Sestrin-2 in HS.

The elevation of MMP-1 and the concurrent decrease in TIMP-1 observed in the HS group are consistent with previous studies highlighting alterations in the protease–antiprotease balance in HS. MMP-1, together with other matrix metalloproteinases such as MMP-2 and MMP-9, contributes to extracellular matrix degradation, whereas TIMP-1 acts as an endogenous inhibitor of matrix metalloproteinase activity [27,28,29]. Accordingly, increased MMP-1 accompanied by reduced TIMP-1 may reflect an altered matrix-remodelling profile in HS. However, no statistically significant association was observed between circulating Nrf2 and TIMP-1 within the HS group (ρ = −0.069, *p* = 0.7360, FDR-adjusted *p* = 0.8899). Therefore, the present data do not support a direct relationship between circulating Nrf2 and TIMP-1 or an Nrf2-mediated regulation of matrix turnover. The MMP-1 and TIMP-1 findings should instead be interpreted primarily as group-level differences requiring confirmation in larger and mechanistically oriented studies.

Asprosin, a fasting-induced glucogenic hormone, has emerged as a metabolic modulator associated with insulin resistance and systemic inflammation. The elevated serum asprosin levels observed in patients with HS may reflect metabolic and inflammatory alterations associated with the disease. However, no statistically significant association was observed between circulating asprosin and Keap1 within the HS group (ρ = −0.003, *p* = 0.9874, FDR-adjusted *p* = 0.9874). Therefore, the present findings do not support a direct relationship between circulating asprosin and Keap1 or a mechanistic link between asprosin and Nrf2–Keap1 signaling. Previous experimental evidence suggests that asprosin may be associated with oxidative stress through mechanisms involving reactive oxygen species production, mitochondrial dysfunction, and pro-inflammatory signaling [17,18,30]; however, these mechanisms were not investigated in the present study. Accordingly, the increased circulating asprosin concentration should primarily be interpreted as an exploratory group-level biomarker difference requiring further mechanistic and clinical validation.

From a pathophysiological perspective, alterations in Nrf2, Keap1, and sestrin-2 levels can be incorporated into a model of impaired cellular stress response. In HS, chronic inflammation drives excessive ROS production by neutrophils, macrophages, and keratinocytes [31]. Under normal conditions, activation of the Nrf2 and sestrin-2 pathways would mitigate this oxidative burden. However, persistent Keap1 upregulation and reduced sestrin-2 levels may inhibit this compensatory mechanism, leading to oxidative DNA damage, lipid peroxidation, and activation of NF-κB-dependent inflammatory cascades. This sustained oxidative and inflammatory environment perpetuates follicular rupture, keratinocyte dysfunction, and immune cell infiltration—the pathological hallmarks of HS [23,32].

Clinically, these findings may have several important implications. First, serum Nrf2, Keap1, and sestrin-2 levels could serve as biomarkers of disease activity, oxidative stress burden, or therapeutic response in HS. Reduced Nrf2 and sestrin-2 levels, together with elevated Keap1, may indicate impaired antioxidant defences and heightened oxidative stress. Therefore, monitoring these biomarkers may help identify patients with increased inflammatory activity. Although pharmacological modulation of Nrf2-related signaling has shown anti-inflammatory and antioxidant effects in chronic inflammatory disorders, including inflammatory skin diseases such as atopic dermatitis and psoriasis, direct evidence in HS remains limited [23,33]. Further experimental and clinical studies are warranted to determine whether targeting this pathway can attenuate oxidative stress, reduce inflammation, and improve clinical outcomes in HS patients. Likewise, strategies aimed at enhancing sestrin-2 expression or activity may represent a novel approach to restoring redox homeostasis and supporting tissue repair [26]. Given the close relationship among sestrin-2, Nrf2 signaling, and oxidative stress regulation, future investigations should explore whether therapeutic modulation of sestrin-2 can influence disease progression and lesion remodeling in HS.

Moreover, the interplay among oxidative stress, protease activity, and metabolic signaling observed in this study underscores the multifactorial nature of HS. A comprehensive therapeutic approach targeting both metabolic and oxidative pathways could yield better outcomes than anti-inflammatory treatments alone. Integrating antioxidant therapy, dietary modification, and biologic agents targeting the TNF-α or IL-17 pathways may offer synergistic benefits.

Strengths and Limitations of the Study: This study has several strengths. To our knowledge, it is among the first to comprehensively evaluate the Nrf2–Keap1–Sestrin-2 axis alongside asprosin and the MMP-1/TIMP-1 balance in patients with hidradenitis suppurativa. Integrating conventional statistical analyses with multivariate approaches, including PCA, PLS-DA, VIP scoring, correlation mapping, and ROC analyses, provided a robust assessment of the molecular profile associated with HS. The highly discriminatory performance of Nrf2 and Sestrin-2 further underscores their clinical relevance. Nevertheless, several limitations should be acknowledged. First, the cross-sectional design precludes causal inference, and the relatively modest sample size may limit generalizability. Second, biomarker measurements were performed only in serum, without tissue-level validation or mechanistic experiments. Third, detailed metabolic and disease-related variables, including BMI, metabolic syndrome, diabetes or insulin resistance, disease duration, IHS4 score, current inflammatory activity, comorbidities, and recently administered nonbiological treatments, were not systematically recorded. Furthermore, differences in gender and smoking history between groups may have introduced residual confounding. Finally, detailed information on inflammatory diseases, active infections, malignancies, metabolic disorders, recent surgeries, systemic medications, antibiotics, corticosteroids, and antioxidant supplements was not systematically available for all participants. Consequently, the influence of remaining confounding factors related to these variables cannot be ignored. Therefore, the observed differences in serum biomarkers should be interpreted cautiously.

## 5. Conclusions

This study showed that patients with hidradenitis suppurativa had altered serum levels of biomarkers related to the Nrf2–Keap1–Sestrin-2 axis and the MMP-1/TIMP-1 balance. Increased serum asprosin, Keap1, and MMP-1 levels, together with decreased Nrf2, Sestrin-2, and TIMP-1 levels, were associated with HS and may reflect alterations in oxidative stress-related and tissue-remodeling processes. However, none of the correlations among these biomarkers within the HS group remained statistically significant after FDR correction. Therefore, the present findings should be regarded as exploratory and do not establish disruption of intracellular pathways, causality, clinical diagnostic utility, or therapeutic relevance. Larger, independent, multicenter, and longitudinal studies are required to confirm these findings and determine their biological and clinical significance.

## Figures and Tables

**Table 1:** Demographic and clinical characteristics of the study population.

Characteristic	Patient	Control	*p*-Value
Age, years, median (IQR)	26.5 (20.25–35.25)	34.0 (28.0–38.25)	0.057
Male sex, *n* (%)	20 (76.9)	9 (45.0)	0.026 *
Female sex, *n* (%)	6 (23.1)	11 (55.0)	
Smoking history, *n* (%)	18 (69.2)	7 (35.0)	0.021 *
Obesity, *n* (%)	9 (34.6)	2 (10.0)	0.082
Family history of HS, *n* (%)	2 (7.7)	—	
Hurley stage I, *n* (%)	5 (19.2)	—	
Hurley stage II, *n* (%)	14 (53.8)	—	
Hurley stage III, *n* (%)	7 (26.9)	—	

* *p* < 0.05.

**Table 2:** Biochemical parameters measured in the serum of patient and control groups.

	Patient	Control	
	Mean ± SD	Mean ± SD	*p* *
Asprosin (ng/mL)	23.47 ± 3.98	21.21 ± 3.38	0.048 *
Keap1 (ng/L)	817.31 ± 115.87	701.18 ± 63.97	0.001 **
Sestrin-2 (ng/mL)	1.87 ± 0.21	2.63 ± 0.64	0.001 **
MMP-1 (ng/mL)	9.28 ± 4.23	6.33 ± 3.99	0.02 *
TIMP-1 (pg/mL)	5.46 ± 1.72	8.26 ± 3.16	0.001 **
Nrf2 (ng/mL)	13.55 ± 3.12	21.15 ± 4.34	0.001 **

* *p* < 0.05, ** *p* < 0.001, Patient Group (*n* = 26), Control Group (*n* = 20).

**Table 3:** Serum Biomarker Correlations in Hidradenitis Suppurativa.

	Nrf2	Keap1	Sestrin-2	Asprosin	MMP-1	TIMP-1
**Nrf2**	—	r = 0.465 *p* = 0.0168 FDR = 0.1258	r = −0.120 *p* = 0.5603 FDR = 0.8899	r = 0.020 *p* = 0.9220 FDR = 0.9874	r = −0.506 *p* = 0.0083 FDR = 0.1246	r = −0.069 *p* = 0.7360 FDR = 0.8899
**Keap1**	r = 0.465 *p* = 0.0168 FDR = 0.1258	—	r = 0.242 *p* = 0.2327 FDR = 0.7851	r = −0.003 *p* = 0.9874 FDR = 0.9874	r = −0.153 *p* = 0.4550 FDR = 0.8899	r = −0.228 *p* = 0.2628 FDR = 0.7851
**Sestrin-2**	r = −0.120 *p* = 0.5603 FDR = 0.8899	r = 0.242 *p* = 0.2327 FDR = 0.7851	—	r = 0.060 *p* = 0.7712 FDR = 0.8899	r = 0.083 *p* = 0.6874 FDR = 0.8899	r = −0.245 *p* = 0.2273 FDR = 0.7851
**Asprosin**	r = 0.020 *p* = 0.9220 FDR = 0.9874	r = −0.003 *p* = 0.9874 FDR = 0.9874	r = 0.060 *p* = 0.7712 FDR = 0.8899	—	r = −0.205 *p* = 0.3141 FDR = 0.7851	r = 0.080 *p* = 0.6976 FDR = 0.8899
**MMP-1**	r = −0.506 *p* = 0.0083 FDR = 0.1246	r = −0.153 *p* = 0.4550 FDR = 0.8899	r = 0.083 *p* = 0.6874 FDR = 0.8899	r = −0.205 *p* = 0.3141 FDR = 0.7851	—	r = 0.128 *p* = 0.5330 FDR = 0.8899
**TIMP-1**	r = −0.069 *p* = 0.7360 FDR = 0.8899	r = −0.228 *p* = 0.2628 FDR = 0.7851	r = −0.245 *p* = 0.2273 FDR = 0.7851	r = 0.080 *p* = 0.6976 FDR = 0.8899	r = 0.128 *p* = 0.5330 FDR = 0.8899	—

## Data Availability

The datasets used during the current study are available from the corresponding author upon request.
